# Free Radical-Mediated Protein Radical Formation in Differentiating Monocytes

**DOI:** 10.3390/ijms22189963

**Published:** 2021-09-15

**Authors:** Ankush Prasad, Renuka Ramalingam Manoharan, Michaela Sedlářová, Pavel Pospíšil

**Affiliations:** 1 Centre of the Region Haná for Biotechnological and Agricultural Research, Department of Biophysics, Faculty of Science, Palacký University, Šlechtitelů 27, 783 71 Olomouc, Czech Republic; renuka.rmanoharan@gmail.com; 2 Department of Botany, Faculty of Science, Palacký University, Šlechtitelů 27, 783 71 Olomouc, Czech Republic; michaela.sedlarova@upol.cz

**Keywords:** U-937 cells, HL-60 cells, phorbol 12-myristate 13-acetate, NADPH oxidase, NOX4, macrophages, protein-centered radicals

## Abstract

Free radical-mediated activation of inflammatory macrophages remains ambiguous with its limitation to study within biological systems. U-937 and HL-60 cell lines serve as a well-defined model system known to differentiate into either macrophages or dendritic cells in response to various chemical stimuli linked with reactive oxygen species (ROS) production. Our present work utilizes phorbol 12-myristate-13-acetate (PMA) as a stimulant, and factors such as concentration and incubation time were considered to achieve optimized differentiation conditions. ROS formation likely hydroxyl radical (HO^●^) was confirmed by electron paramagnetic resonance (EPR) spectroscopy combined with confocal laser scanning microscopy (CLSM). In particular, U-937 cells were utilized further to identify proteins undergoing oxidation by ROS using anti-DMPO (5,5-dimethyl-1-pyrroline N-oxide) antibodies. Additionally, the expression pattern of NADPH Oxidase 4 (NOX4) in relation to induction with PMA was monitored to correlate the pattern of ROS generated. Utilizing macrophages as a model system, findings from the present study provide a valuable source for expanding the knowledge of differentiation and protein expression dynamics.

## 1. Introduction

Macrophages from the innate immune system serve as the front line of human defense against pathogens, thus their dysfunction leads to clinical symptoms, such as septic shock or chronic inflammatory diseases [1]. Regulatory mechanisms involved in the process of hematopoietic cell differentiation are not completely known, and disturbances in their gene network can result in several neoplastic disorders [2]. Macrophage differentiation from monocytes depends on the acquisition of microenvironmental signals, and is characterized by a change in cell morphology, downregulation of membrane proteins, and increased cytoplasmic complexity [3,4].

To date, several models have been employed to study the modulation associated with monocytes and macrophages during inflammatory diseases. Though peripheral blood mononuclear cells (PBMCs) remain a widely accepted model, technical disparities in their handling and donor-to-donor variations make human leukemic cell lines U-937 (monocytic) and HL-60 (promyelocytic) the most widely employed model for investigating cell differentiation and its subsequent functioning [5]. It displays numerous properties of immature monocytic cells over THP-1, due to its blood leukemic origin. These immature cells can be induced by various stimuli, such as vitamin D3, 12-O-Tetradecanoyl Phorbol-13-Acetate (TPA), Phorbol 12-Myristate 13-Acetate (PMA), and retinoic acid along the monocytic/macrophage pathway, with outcome based on given stimulus and duration of differentiation [6,7,8,9,10,11]. A diester of phorbol, Phorbol 12-myristate 13-acetate stimulates cells to undergo monocytic differentiation, thereby acquiring typical phenotypic and functional characteristics of macrophages [7,12,13]. Activated macrophages, endothelial cells, and leukocytes produce reactive oxygen species (ROS) which are in turn implicated in a plethora of pathological processes in the human body, including asthma, diabetes, and atherosclerosis [14].

Ubiquitous in nature, ROS are reactive molecules and thus extremely short half-lived [15,16,17,18,19,20]. As a part of defense mechanisms, ROS are produced during the respiratory burst of phagocytes to cope with invading pathogens. Reactive oxygen species regulate multiple cellular processes, such as growth, differentiation, apoptosis, and gene expression cascades [21]. The rate of an oxidant reaction with biological molecules primarily depends on its concentration and reaction rate constant. Since proteins constitute nearly 70% of cell dry mass, oxidative damage within the biological system is most likely confined to the protein modification. Protein oxidative damage generated upon these biomolecules is generally initiated by extraction of a weakly bonded hydrogen atom to form protein radicals (carbon-centered and oxygen-centered) as transient intermediates [22,23,24,25,26,27]. These reactive intermediates can ultimately result in fragmentation, aggregation, or post-translational protein modifications. Among the spin trap compounds, 5,5-Dimethyl-1-pyrroline N-oxide (DMPO) possesses the best pharmacokinetics properties with less toxicity, making it a primary choice for investigating radical species in biological systems [22,28,29,30,31,32]. ROS-mediated oxidation produces carbonyl groups, namely aldehydes and ketones, on protein side chains that serve as biomarkers for protein oxidative stress [33]. Intracellular enzymatic sources and reduced nicotinamide adenine dinucleotide phosphate (NADPH) oxidases, also referred as NOX, contribute equally to ROS/oxidation products generation in monocytes, with the latter predominating with subunits that are localized both in plasma membrane as well as in cytosol [34,35]. Deciphering the mechanism involved in NADPH oxidase activation can aid in designing strategies for selective inhibition of ROS by differentiating monocytes. Apocynin, a naturally occurring methoxy substituted catechol, was used as an inhibitor of NADPH oxidase to evaluate its expression profile upon differentiation with PMA. The inhibitory action of apocynin is quite confined towards ROS-mediated inflammation sites, as it requires ROS and myeloperoxidase (MPO) for its activation [36]. Among seven known NOX subtypes, NOX4 endures to be the most extensively distributed isoform with downstream expression production of H_2_O_2_. With its indispensable role in ROS generation and signal transduction, its abnormal expression or overactivation may lead to tissue damage [37].

From our previous studies, it has been evident that cell induction with PMA alone produces a cascade of oxidant species via nitric oxide synthase and myeloperoxidase [6]. It is highly recommendable to determine the most appropriate differentiation protocol as this can significantly impact their response to various innate stimuli. Considering these facts, our present study focuses in more detail on U-937 differentiation with PMA and its resulting production of ROS. The study aids us to understand the involvement of ROS in the differentiation process and evaluates NADPH oxidase 4 (NOX4) expression profile during the transition from monocytes into macrophages [38] in the presence and absence of apocynin by western blot analysis. Reactive oxygen species generation in differentiated cells was confirmed with electron paramagnetic resonance (EPR) spectroscopy using spin trapping and the same has been established with confocal imaging using hydroxyphenyl fluorescein (HPF). HPF show limited reactivity and higher resistance to light-induced oxidation and is nonfluorescent until it reacts with HO^●^, but its reactivity with peroxynitrite anion and hypochlorite anion cannot be completely ruled out (datasheet: Molecular Probes, H36004), which is also a shortcoming of most fluorescent compounds for specific detection of ROS [39]. Free radical-mediated protein oxidation in PMA differentiated U-937 cells was investigated using the immuno-spin trapping technique. Formation of DMPO nitrone adducts was detected with the use of anti-DMPO antibody. Results from this study validate the optimum differentiation protocol to achieve a macrophage-like phenotype with PMA in U-937 and HL-60. It can be extended further to compare PMA mediated proteome changes along the differentiation process that aids in protein modification and expression pattern changes.

## 2. Results

### 2.1. Cell Differentiation by Phorbol 12-Myristate 13-Acetate

U-937 cells are widely utilized to study the responses in differentiating macrophages. According to studies carried so far, PMA can aid in cell differentiation to a macrophage via Protein kinase C (PKC) activation. Since there exists a wide range of PMA treatment methodologies in terms of concentration and incubation time, our present study started with the protocol optimization. We aimed at optimizing PMA mediated differentiation conditions in two (pro-)monocytic cell lines, explicitly U-937 and HL-60, and further proteomic characterization during its progression from monocytic to macrophage-like cells were carried out in U-937 cell lines to determine oxidative stress-mediated protein oxidation.

**Figure 1:**
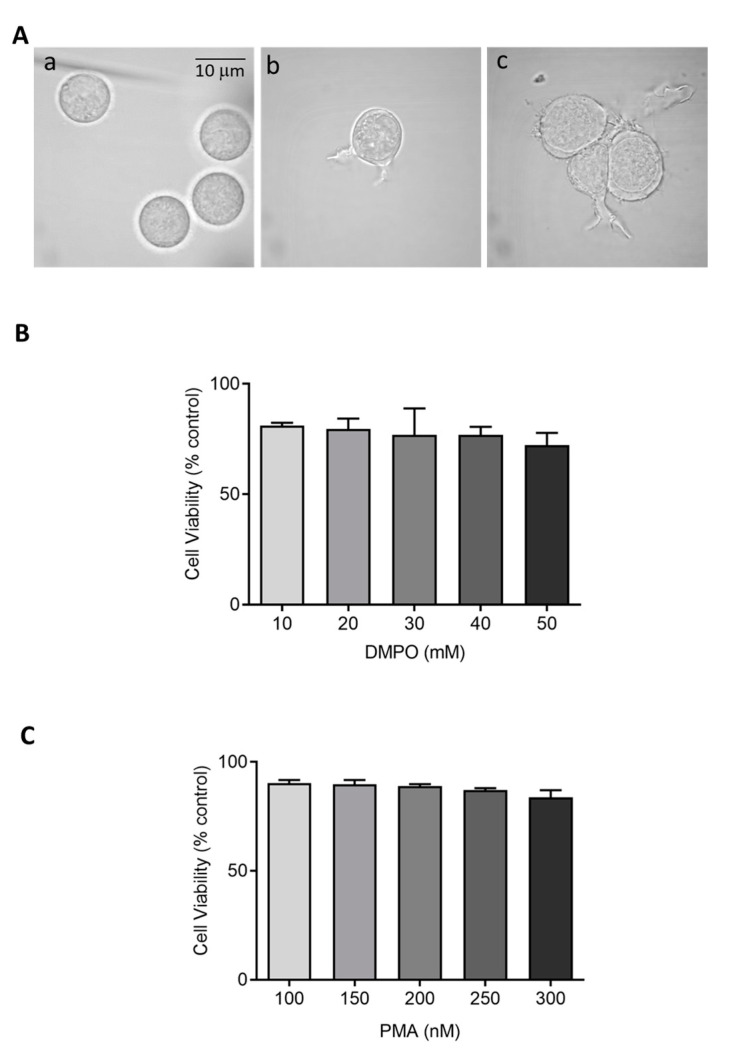
(**A**) Morphology of U-937 cells in the absence (a) and presence of 150 nM PMA for 48 h (b) and 72 h (c) post incubation at 37 °C. (**B**,**C**) Cell viability (%) of U-937 cells post incubation with DMPO and PMA at varying concentration. The data are represented as mean ± SE.

**Figure 2:**
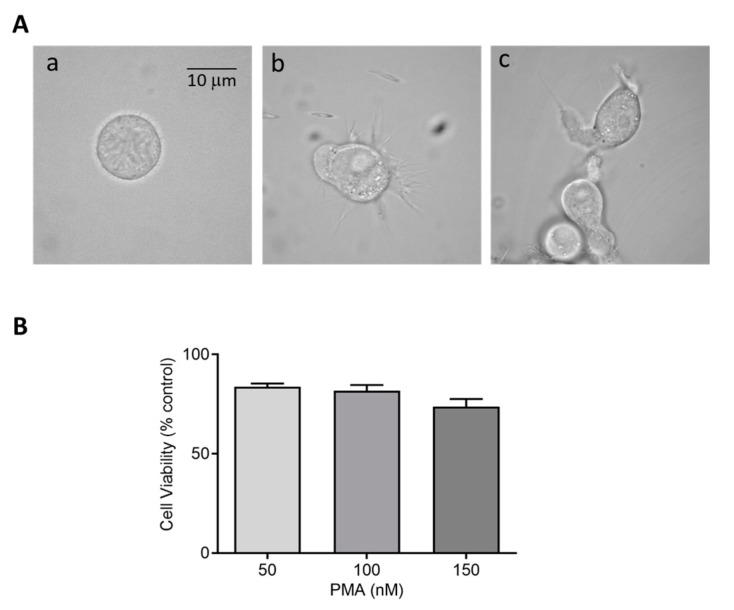
(**A**) Morphology of HL-60 cells in the absence (a) and presence of 50 nM PMA for 48 h (b) and 72 h (c) post incubation at 37 °C. (**B**) Cell viability (%) of HL-60 cells post treatment with 50 nM, 100 nM, and 150 nM PMA. The data are represented as mean ± SE.

Herein, we selected different PMA concentrations, 150 and 250 nM for U-937 cells and 25–100 nM for HL-60 cells, along with two different incubation periods of 48 h and 72 h, to achieve monocytic to macrophage-like cells. Microscopic analysis revealed a distinct pattern of differentiation in cells treated with PMA concentrations compared to round floating cells observed in untreated monocytes. Concerning U-937, a dosage of 150 nM and 250 nM PMA for 72 h incubation provided the most efficient differentiation without cell death (Supplementary Figure S1). Post induction, cells were maintained further for a period of 24 h in serum-free media as a resting phase. To validate the finding, the effect of differentiation inducer (PMA, 25–100 nM) was also measured on HL-60 cells showing similar effects. For HL-60, a PMA concentration of 50 nM differentiated for a period of 48 h turned out to be an efficient differentiation condition to achieve enhanced cell adherence and granularity. These data confirm that differentiation initiated by PMA varies among cell lines based on its concentration and period of incubation. Figure 1 and Figure 2 exemplify the altered morphology of monocyte-derived macrophages post PMA treatment. Cells exhibit an increased cellular adherence, volume, and granularity with the presence of pseudopodia. Z-stack series of 10 optical sections (distance 0.48 µm) through a differentiated U-937 cell treated with PMA is included as Supplementary Figure S2.

**Figure 3:**
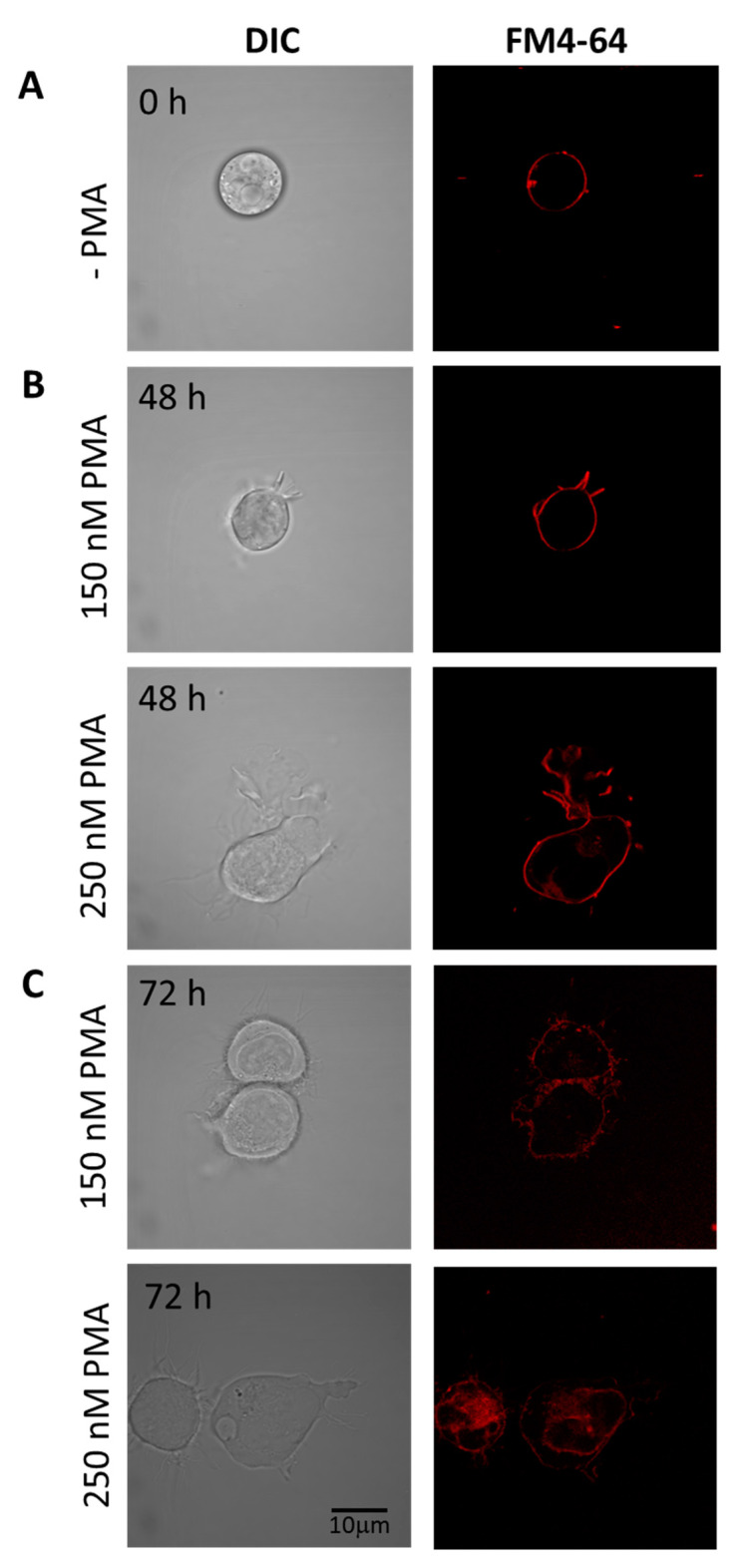
Staining of U-937 cells with FM4-64 (5 min, RT) to check membrane integrity in non-differentiated (**A**) and differentiated cells for 48 h (**B**) and 72 h (**C**). In both B and C, the upper panel shows U937 cells treated with 150 nM PMA, while the lower panels are cells treated with 250 nM PMA. Individual channels for DIC and FM4-64 (red signal) are presented.

**Figure 4:**
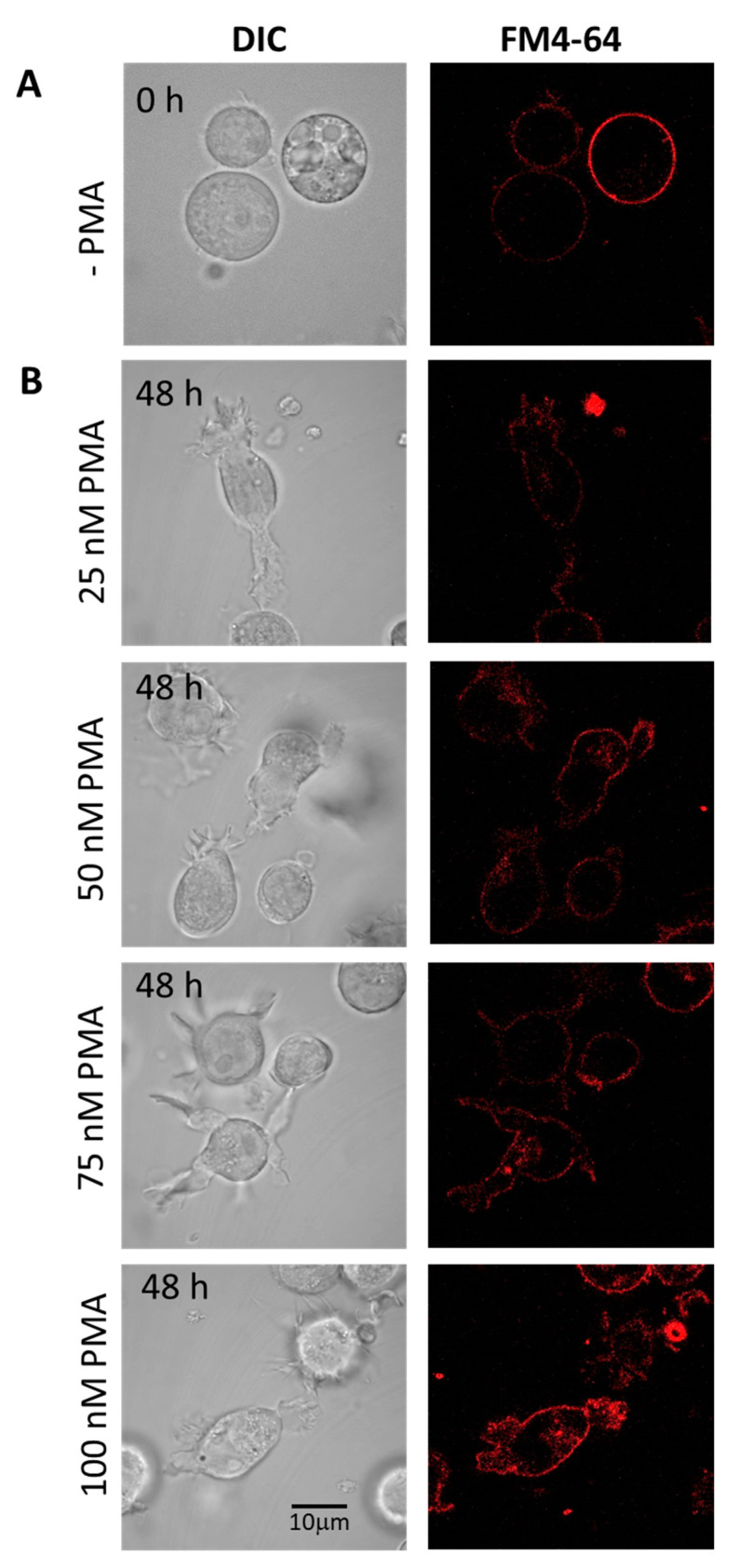
Staining of HL-60 with FM4-64 (5 min, RT) visualizing membrane integrity in non-differentiated (**A**) and 48 h differentiated (**B**) HL-60 cells. In B, the panels show cells treated with 25 nM, 50 nM, 75 nM, and 100 nM PMA. Individual channels for DIC and FM4-64 (red signal) are presented.

Cells differentiated under 48 h (Figure 3B) and 72 h (Figure 3C) experimental conditions are presented individually, with upper panel holding U-937 cells treated with 150 nM PMA and lower panel with 250 nM PMA. HL-60 cells treated with four different concentrations of PMA and corresponding membrane integrity has been shown in Figure 4. Panels A in both Figure 3 and Figure 4 represent non-differentiated control cells of U-937 and HL-60, respectively.

### 2.2. Cell Viability or Proliferation Assays

Cytotoxic effects of perturbing factors should be investigated via a cell viability test. Quantitative estimation of viable cells exposed to varying concentrations of DMPO (U-937) and PMA (U-937 cells; HL-60 cells) was carried out using MTT assay. Viable cells in mitochondria reduce MTT to formazan which is later solubilized, and its absorbance recorded at 570 nm. The value obtained by this colorimetric method thus corresponds to metabolically active cells. To study the effect of DMPO (10–50 mM) and PMA (100–300 nM) induced cytotoxicity, U-937 cells were treated with PMA for 72 h and DMPO for 24 h. A loss of cell viability (∼30%) was observed when U-937 cells were treated with an increasing concentration of DMPO (Figure 1B) and ∼20% with PMA (Figure 1C). Thus, 10 mM DMPO and PMA in concentration of 150 and 250 nM were used for further studies. Similarly, PMA (50–150 nM) induced cytotoxicity was evaluated in HL-60 cells. Figure 2B illustrates that more than 75% cells retain viability upon treatment with PMA and the concentration of 50 nM was used for further studies of differentiation processes.

### 2.3. Hydroxyl Radical Imaging during Cell Differentiation

**Figure 5:**
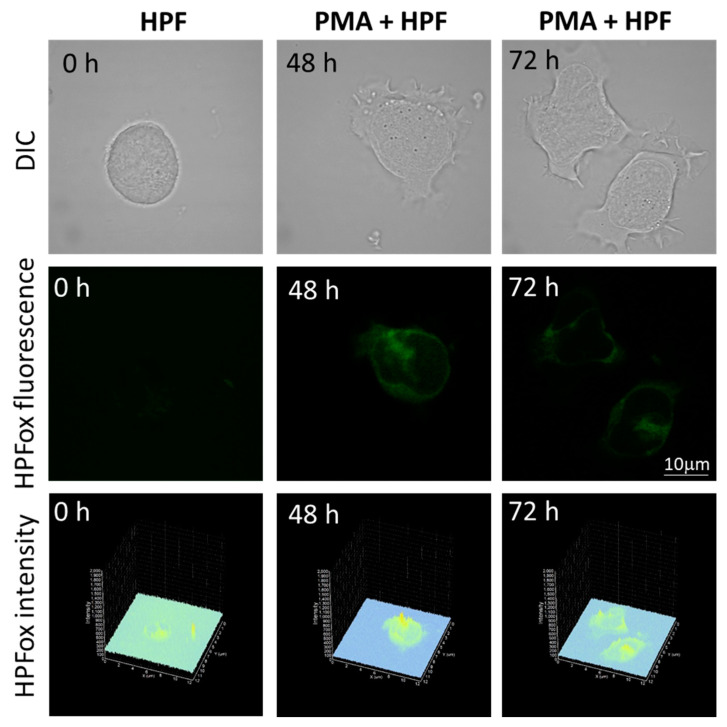
Hydroxyl radical imaging. U937 cells were incubated with 1 μM HPF for 12 h in the absence (**left panel**) and presence of 250 nM PMA for 48 h (**middle panel**) or for 72 h (**right panel**). Images from DIC channel and HPF-ox fluorescence (green signal) are presented. The lower panel shows the integral distribution of the signal intensity within the sample (*Z*-axis represents the levels of brightness for each pixel, ranging between 0 and 2000).

**Figure 6:**
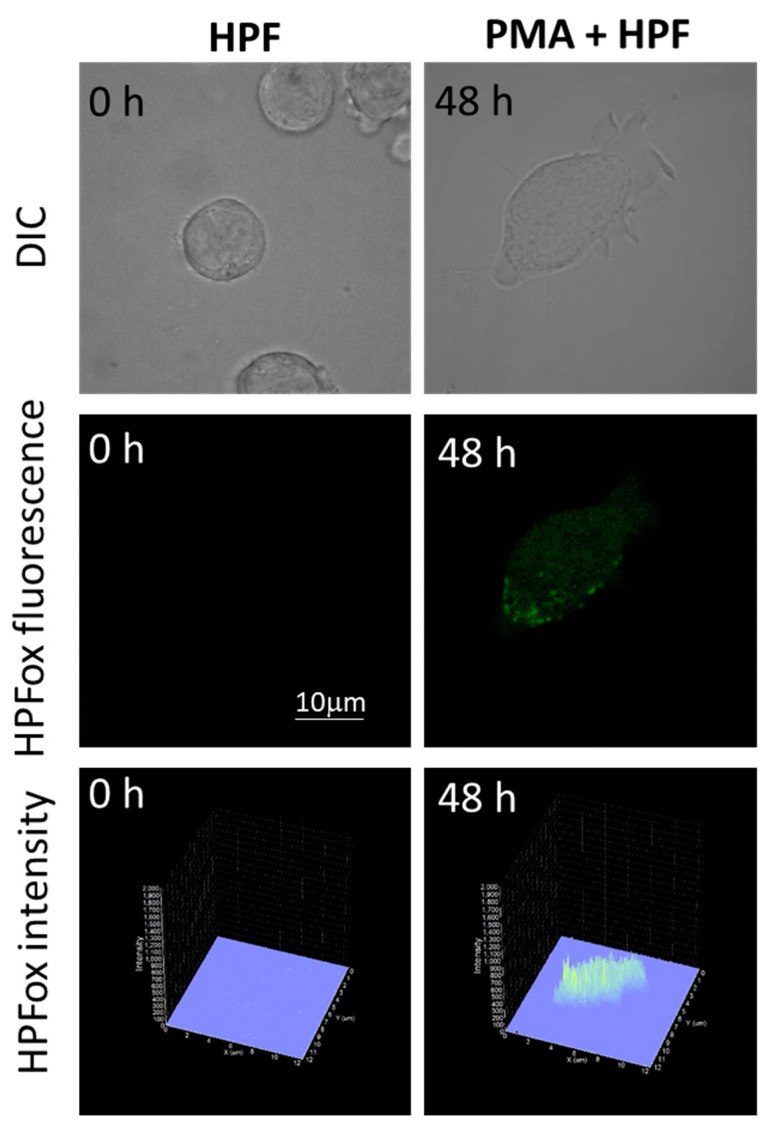
Hydroxyl radical imaging in HL-60 cells. The cells were incubated with 1 μM HPF for 12 h in the absence (**left panel**) and presence (**right panel**) of 50 nM PMA. The incubation in PMA was done for 48 h. Images from DIC channel and HPF-ox fluorescence (green signal) are presented. The lower panel shows the integral distribution of the signal intensity within the sample (*Z*-axis represents the levels of brightness for each pixel, ranging between 0 and 2000).

The subsequent generation of HO^●^ following superoxide anion radical (O_2_^●−^) production by differentiating U-937 and HL-60 cells was validated by confocal microscopy using HPF fluorescent probe. In the presence of HPF, U-937 cells treated with 250 nM of PMA for 48 h exhibited an increase in HPFox fluorescence and it was significantly enhanced after 72 h (Figure 5, middle panel and lower panel). Negligible fluorescence was observed with control cells (Figure 5, left panel). In the case of HL-60 cells, HPFox fluorescence was measured in the presence of 50 nM PMA for 48 h, which showed enhanced fluorescence compared to control (Figure 6). These results clearly indicate that the differentiation stage and HO^●^ formation are closely interlinked. To validate these findings, EPR spectroscopy using spin trapping was further utilized as described in the next section.

### 2.4. Detection of Hydroxyl Radical Formation by EPR Spin Trapping Spectroscopy

**Figure 7:**
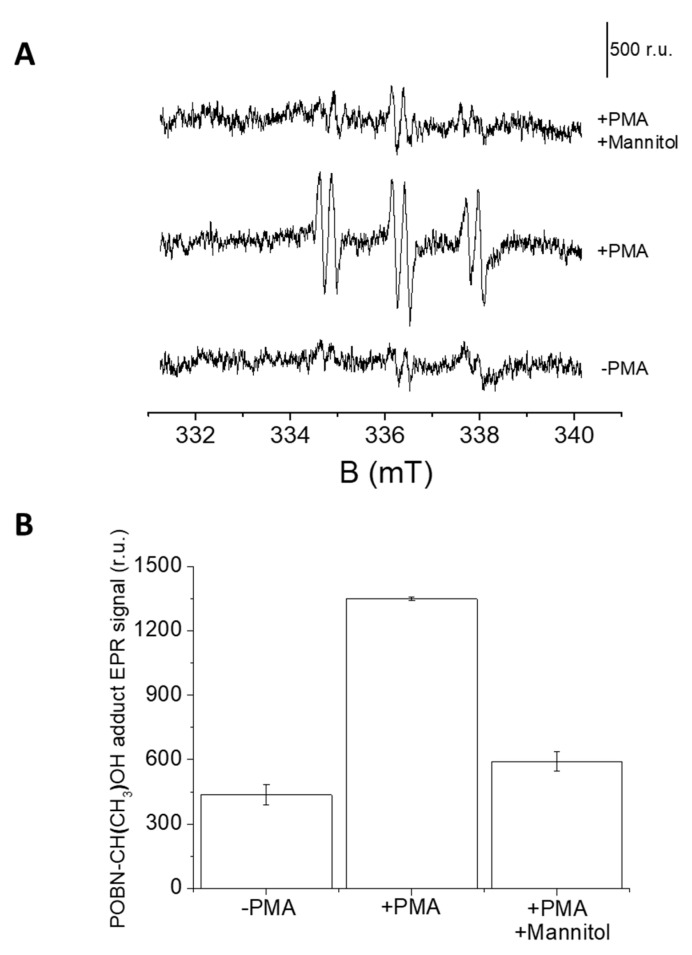
(**A**) PMA induced POBN-CH(CH_3_)-OH adduct EPR spectra. U-937 cells were incubated with 50 mM POBN in the absence (lower trace) or presence of 50 μM PMA (middle trace) and 50 μM PMA + 5 mM mannitol (upper trace). Vertical bar represents 500 relative units. (**B**) Bar chart representing values +/- SD of EPR spectra measurements (*n* = 3).

In U-937 cells, the formation of HO^●^ was measured by EPR spectroscopy using a POBN/ethanol spin trap system. No or negligible intensity of EPR signal was observed in control U-937 cells (Figure 7A, lower trace), whereas treatment with 50 μM PMA for 20 min resulted in the formation of the α-hydroxyethyl radical adduct of POBN (POBN-CH(CH_3_) OH adduct) EPR signals (Figure 7A, middle trace). It can be seen that the addition of mannitol, which is a known HO^●^ scavenger, prior to PMA treatment led to significant suppression of EPR signal (Figure 7A, upper trace and Figure 7B). The current observation validates the formation of HO^●^ under PMA induced respiratory burst.

### 2.5. Protein-Centered Radicals Detection from U-937 Cells

**Figure 8:**
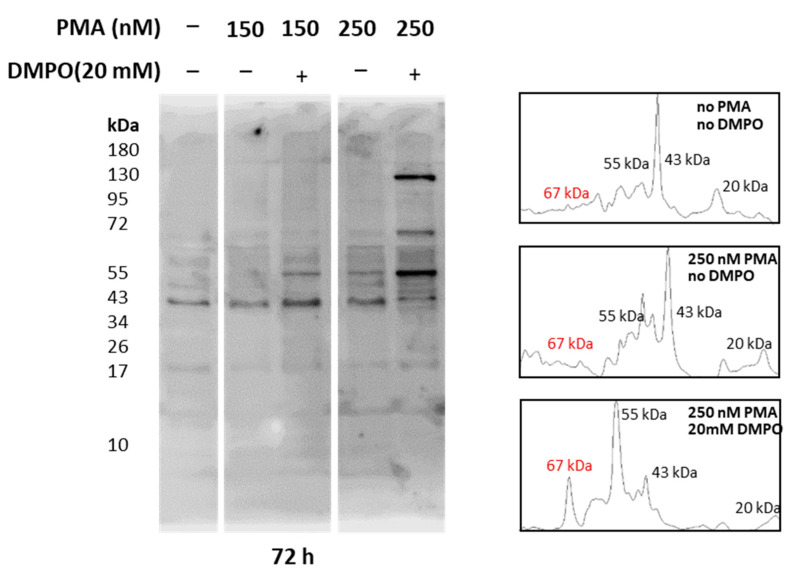
Identification of protein-DMPO nitrone adducts in U-937 cells differentiated with PMA. Western blot analysis of protein-DMPO nitrone adducts in whole-cell homogenates treated with 150 nM and 250 nM PMA for 72 h (**left panel**) and their corresponding densitograms (**right panel**).

Whole-cell homogenate extracted from U-937 cells treated with PMA and DMPO was separated using SDS PAGE and bands were visualized with Coomassie blue staining. As entire cell homogenate was used for analysis, bands of 17 kDa, 35 kDa, 40 kDa, 43 kDa, 55 kDa, 67 kDa, and 130 kDa were observed (Supplementary Figure S3). Reactive oxygen species generation from cells differentiated with PMA has been proven above with EPR spectroscopy and confocal microscopy (Figure 5, Figure 6 and Figure 7), while intermediate species, namely protein-centered radicals formed by these ROS species, were confirmed with spin trap DMPO (nitrone adducts) [40,41]. To identify proteins tagged with DMPO, total homogenates from cells treated with PMA and DMPO were analyzed using an anti-DMPO western blot analysis. Total cell homogenate blots incubation with a specific antibody revealed more than 10 bands (Figure 8). Upon differentiation, expression of 130 kDa protein became evident with an increasing concentration of PMA, and protein bands of 55 kDa and 67 kDa were enhanced comparatively with respect to controls. Densitograms illustrating protein expression in comparison to DMPO controls are included in Supplementary Figures S4 and S5.

### 2.6. Effect of Apocynin on Hydroxyl Radical Formation and NADPH Expression

**Figure 9:**
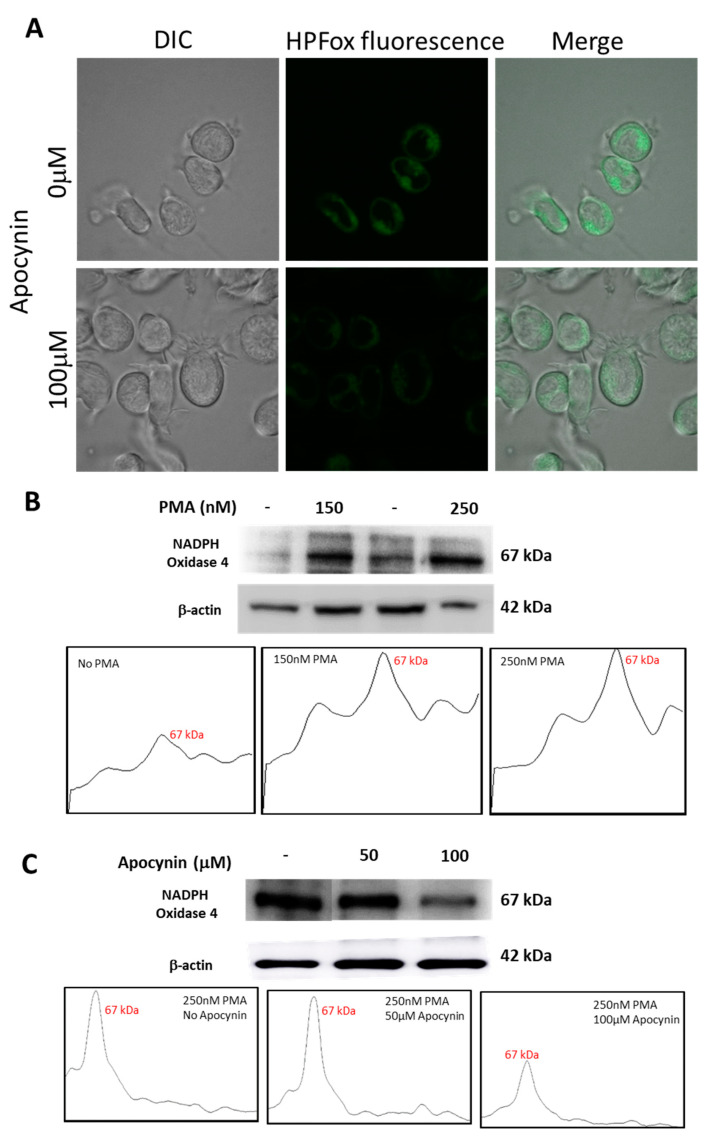
(**A**) Hydroxyl radical imaging using CLSM. U-937 cells were incubated with 1 μM HPF and 250 nM PMA in the absence (upper panel) and presence (lower panel) of 100 µM apocynin. Images from DIC channel and HPF-ox fluorescence (green signal) are presented. (**B**) Western blot analysis of NADPH oxidase 4 expression in whole-cell homogenate samples treated with 150 nM and 250 nM of PMA for 72 h and their corresponding densitograms. (**C**) Western blot analysis of NADPH oxidase 4 expression in 250 nM PMA differentiated whole cell homogenates pre-treated with 50 µM and 100 µM apocynin and their corresponding densitograms.

U-937 cells treated with apocynin prior to differentiation with PMA displayed a reduction in HO^●^ generation and the same was validated using HPF fluorescent probes by confocal laser scanning microscopy. It can be seen from Figure 9 that HPFox fluorescence was higher in control samples compared to apocynin treated samples. The intracellular HO^●^ generation was comparatively higher in the control sample (Figure 9A, upper panel) treated with 250 nM PMA alone and found to be partially suppressed in the presence of 100 μM apocynin (Figure 9A, lower panel). Activation of NADPH oxidase enhances ROS production resulting in oxidative stress and subsequent dysfunctional regulation of signal transduction cascades leading to pathological changes [42,43]. From Figure 9B, it is apparent that expression of NOX4 becomes evident with increasing concentration of PMA. The corresponding densitograms are shown as a part of Figure 9B. Nevertheless, incubation with apocynin prior to differentiation with PMA reduces NOX4 expression (Figure 9C). The differences in NOX4 expression patterns with increasing concentrations of apocynin are represented as densitograms in Figure 9C. Taken together, our data showed that NOX4 expression remained upregulated in the PMA induced differentiating macrophage cell line, while treatment with apocynin downregulated NOX4 in the in vitro system. However, differences in inhibitory pattern can be attributed to the availability of myeloperoxidase, which initiates active apocynin dimer conformation to oxidize NOX [44]. Variance in NOX4 expression pattern upon treatment with different concentrations of PMA and apocynin is presented as Supplementary Figure S6.

## 3. Discussion

Selection of characteristic cell line, and development and usage of experimental conditions that modulate cellular activity remain important key factors in ensuring consistent and reproducible results. The human leukemia cell lines U-937 and HL-60 represent a commonly employed representative model to elucidate mechanisms of monocytic or myelocytic differentiation. Despite its wide acceptance as a suitable in vitro model, a standardized protocol for differentiation of pro monocytes into macrophages using stimuli such as PMA exists. In general, macrophages represent a dynamic cell population whose functional and phenotypic characteristics varies significantly based on the nature of stimuli used and environmental factors in which they mature. Lack of a defined protocol poses a significant impact on results interpretation [45,46]. Considered as activating stimulus, PMA induces differentiation of macrophages with almost similar phenotypes, such as cell morphology, surface marker expression, and cytokine production [46]. To illustrate the course of monocyte to macrophage differentiation, two different conditions were employed in our study. The treatment condition included usage of high concentration of PMA (induction phase), followed by resting period. Results obtained from confocal microscopy display better differentiating macrophage-like phenotype with condition B compared to condition A. Cytotoxicity assays were carried out to validate the stimulus and spin trap concentration used in this study, and no significant cytotoxicity was caused either by PMA or DMPO as assessed by mitochondria function (MTT assay).

In the EPR spin trapping data obtained using the mannitol, which is a potent HO^•^ scavenger, the POBN-CH(CH_3_)-OH adduct EPR signal was found to be significantly suppressed, which indicates that HO^•^ is predominantly formed during respiratory burst induced with PMA (Figure 7A). The mannitol-insensitive POBN-CH(CH_3_)-OH adduct EPR signal (~15%) is likely due to specificity of mannitol or inclined affinity of HO^●^ towards POBN compared to mannitol. It has been previously proposed that HO^●^ detected in the cells are formed by the dismutation of O_2_^●−^ to H_2_O_2_ and subsequently forming HO^●^ in the presence of metal ions [6].

Inflated ROS production, oxidized proteins accumulation, and peroxidases expression are considered to be important markers of macrophage stress at sites of inflammation [47]. Our findings demonstrate protein-centered radical formation as a result of ROS dependent proteotoxic stress caused by varying doses of PMA in U-937 cells. In general, one-electron oxidation reaction of protein involving peroxidases, redox active metals, and peroxynitrite can result in formation of protein-centered radicals. Cell permeable spin trap DMPO reacted with oxidized protein radicals and formed DMPO nitrone adducts. The adducts thus formed were resolved with SDS-PAGE, transferred onto nitrocellulose membrane, and identified with immunoblotting analysis using anti-DMPO antibodies.

Among the reported source of ROS producing enzyme complexes, NOX serve as a key source of ROS in many conditions. Recent discoveries propose a close association between increased ROS production and upregulated NOX4 expression [48]. Thus, herein we reported the expression pattern of NOX4 in response to stimuli such as PMA and apocynin. Known to be the most well characterized NADPH oxidase inhibitor, apocynin acts by inhibiting NADPH oxidase assembly in monocytes, neutrophils, and endothelial cells [49]. Exposure of U-937 cells to 150 nM of PMA for 48 h resulted in distinct NOX4 expression and it increased progressively with concentration and time until 250 nM for 72 h. However, treatment with apocynin subsides NOX4 expression in differentiating macrophage cell lines. Studies must be carried out to study downstream and upstream regulation of this particular NOX4 in directing free radical-mediated proteomic stress in activated macrophages.

## 4. Materials and Methods

### 4.1. Reagents and Antibodies

Human leukemia cell lines (U-937 and HL-60) were purchased from American Type Culture Collection (ATCC; Rockville, MD, USA). Phorbol 12-myristate 13-acetate was obtained from Sigma Aldrich (St. Louis, Missouri, United States). 5,5-Dimethyl-1-pyrroline N-oxide was obtained from Dojindo Molecular Technologies (Rockville, MD, USA). Recombinant anti-NADPH oxidase 4 antibody and rabbit polyclonal Anti-DMPO Nitrone adduct antibody were purchased from Abcam (Cambridge, CB2 0AX, UK). Goat polyclonal anti-rabbit IgG conjugated with horseradish peroxidase (HRP) were obtained from Bio-Rad (Hercules, CA, USA). Cell culture media and antibiotics were procured from Biosera (Nuaille, France). Protease and phosphatase inhibitors were obtained from Roche (Mannheim, Germany).

### 4.2. Cell Culture and Treatment regime

U-937 and HL-60 cells were cultured in RPMI -1640 medium supplemented with 10% fetal bovine serum, 1% (*v*/*v*) antibiotic solution (penicillin/streptomycin), and L-glutamine (0.3 g/L). Cells were subcultured at 37 °C in a humified atmosphere aerated with 5% CO_2_. For differentiation studies, cells were treated with different doses of PMA; 150 or 250 nM for U-937 cells, 25 to 100 nM for HL-60 cells, with time intervals (induction phase). Two different treatment regimens were followed for U-937 cells wherein condition I cells (1 × 10^6^ colony forming unit (CFU)/mL) were exposed to 150 and 250 nM of PMA for 48 h and in condition II for 72 h. For HL-60 cells, PMA treatment was performed at 25 nM, 50 nM, 75 nM, and 100 nM for 48 h (otherwise stated in figure legend).

Cells were washed with phosphate buffer saline (PBS) (pH 7.4) to remove residual PMA and non-adherent cells. Cell density was estimated using TC20 Automated Cell Counter (Bio-Rad Laboratories, Hercules, CA, USA). Cell morphology was examined on slides using Fluorview 1000 confocal unit attached to IX80 microscope (Olympus Czech Group, Prague, Czech Republic), in the following text abbreviated as CLSM. To determine the role of NADPH oxidase, specifically NOX4 in the monocyte to macrophage differentiation process, U-937 cells were pre-treated with apocynin (50–100 μM, 1 h) before the addition of PMA. To characterize protein centered radicals formed during the process of differentiation, U-937 cells were incubated with 10 mM DMPO for 24 h in serum free media.

### 4.3. Cell Proliferation Assay

Metabolic activity of U-937 cells as well as HL-60 cells upon treatment with PMA and DMPO were examined by MTT assay kit (Abcam, Cambridge, UK). Briefly, U-937 cells were grown (1 × 10^4^ CFU/mL) in 96 well microplates and treated with varying concentrations of DMPO (10–50 mM) and PMA (100–300 nM) for 24 h. Similarly, HL-60 cells were treated with varying concentrations of PMA (50–150 nM) for 24 h. Post-treatment, cells were incubated with MTT for 3 h at 37 °C, and absorbance was read at 590 nm.

### 4.4. Cell Membrane Integrity

Cells were grown on six-well cell culture plates (Greiner Bio One Ltd., Stonehouse, UK). Post-treatment with PMA both U-937 and HL-60 cells were washed with PBS and membrane integrity was monitored under tested experimental conditions by staining cells with amphiphilic styryl dye FM4-64 (15 μM, 5 min, RT). FM4-64 was visualized in cells placed on slides by CLSM using excitation by a 543 nm He-Ne laser and 655–755 nm emission filter. Imaging was combined with a transmitted light detection module with 405 nm diode laser excitation and Nomarski/differential interference contrast (DIC) filters to follow morphology of low-contrast cells.

### 4.5. Hydroxyl Radical Imaging Using Confocal Laser Scanning Microscopy (CLSM)

Intracellular ROS production was examined using confocal laser scanning microscopy, wherein both PMA differentiated cells and control cells were incubated with 1 µM Hydroxy Phenyl Fluorescein (HPF) in HEPES buffer (pH 7.2). HPF, which is almost non-fluorescent in neutral solutions, reacts with highly reactive oxygen species, namely hydroxyl radical, resulting in increased fluorescence intensity. Excitation of samples was performed using a 488 nm line of an argon laser and the HPFox fluorescence was detected by a 505–605 nm filter.

### 4.6. Hydroxyl Radical Detection Using EPR Spin Trapping Spectroscopy

Formation of HO^●^ by differentiating U-937 cells was measured by EPR spectrometer, MiniScope MS400 (Magnettech GmbH, Berlin, Germany). U-937 cells were treated with 50 μM PMA in absence or presence of 5 mM mannitol as a scavenger and incubated for 20 min at 37 °C and 5% CO_2_. PMA differentiated cells were incubated with 50 mM POBN containing 170 mM ethanol for 20 min at 37 °C. EPR spectra were recorded under the following parameters: microwave power (10 mW), modulation amplitude (1 G), modulation frequency (100 kHz), sweep width (100 G), scan rate (1.62 G s^−1^). Incubation steps were skipped for baseline recording.

### 4.7. Whole-Cell Protein Lysate Preparation

To carry out proteomic analysis, U-937 cells were cultured and differentiated as mentioned above. Both PMA-treated and control cell samples were incubated in serum-free media in the presence of 10 mM DMPO. Post incubation, cells were harvested by centrifugation and washed with ice-cold PBS (pH 7.4) to remove residual media. Resulting cell pellets were resuspended in lysis buffer (150 mM NaCl, 50 mM Tris (pH 8.0), 0.5% sodium deoxycholate, 0.1% SDS, 1% NP-40, and 1% (*v*/*v*) complete protease inhibitor cocktail) and subjected to sonication (four cycles with 20 sec on and 10 off). The processed homogenate was subjected to centrifugation at 12,000–14,000 rpm and the collected supernatant fraction was quantified using a Pierce BCA protein assay kit (Thermo Fisher Scientific, Paisley, UK).

### 4.8. Western Blot Analysis of Nitrone Adducts

U-937 cell homogenates were mixed with 5× Laemmli sample buffer and 100 mM 2-Mercaptoethanol. Following denaturation, protein samples (20 µg/lane) were separated on 10% SDS gels and transferred onto PVDF membrane using Trans-Blot Turbo Transfer System (Bio-Rad, Hercules, CA, USA). Membranes were blocked with 5% BSA in Tris buffered saline (TBS) (pH 7.4) along with 0.1% Tween 20 for 1 h at room temperature. Blocked membranes were probed with rabbit polyclonal anti-DMPO nitrone adduct antibody overnight at 4 °C, followed by incubation with HRP-conjugated anti-rabbit secondary antibody (1:10,000 dilution) for 1 h at room temperature. Immunocomplexes thus formed were developed with Immobilon Western Chemiluminescent HRP Substrate (Sigma Aldrich, GmbH, Germany) and recorded with Amersham Imager 600 (GE Healthcare, UK). Dilution of 1:5000 was used for both anti-DMPO and β-actin antibodies.

To delineate the role between NOX4 and ROS generation in differentiating cells, we used Anti-NADPH oxidase 4 specific antibodies from Abcam. Densitometry analysis of western blots were performed using Image J, public domain software (Bethesda, MD, USA) provided by the National Institute of Mental Health, United States. Peaks in the densitogram corresponds to quantification of protein band intensity.

## 5. Conclusions

Human leukemia cell lines U-937 and HL-60 serves as a well-documented macrophage model system, and protocols to achieve macrophage differentiation vary among reported literature. Most of the studies carried out so far primarily focused on characterizing transcriptional profiling, and it is necessary to study the impact of differentiation at the proteome level, as effect to any external stimuli is a global response. To demonstrate it, we employed two variants of differentiation protocols that include a higher concentration of stimulant and incubation period. In general, macrophages at the site of inflammation were characterized by excess ROS production and accumulation of oxidized proteins. Results obtained correlate well with expected macrophage-like phenotype, wherein ROS generated were confirmed with EPR spectroscopy confocal microscopy. Immunoblotting with anti-DMPO antibodies reveals proteins had undergone oxidation mediated by ROS produced within the system. This study must be extended further to study and characterize specific proteins tagged with DMPO during differentiation and their possible role in disease and inflammation.

## Data Availability

No new data were created or analyzed in this study. Data sharing is not applicable to this article.

## Supplementary Materials

The Supplementary Materials are available online at https://www.mdpi.com/article/10.3390/ijms22189963/s1.

## Abbreviations

ROS: reactive oxygen species; EPR spectroscopy: electron paramagnetic resonance spectroscopy; CLSM: confocal laser scanning microscopy; PMA: phorbol 12-myristate 13-acetate; RIPA: radioimmunoprecipitation assay buffer; CFU: colony-forming unit.

## References

[1] Luan Y.-Y., Dong N., Xie M., Xiao X.-Z., Yao Y.-M. (2014). The Significance and Regulatory Mechanisms of Innate Immune Cells in the Development of Sepsis. J. Interf. Cytokine Res..

[2] Liggett L.A., Sankaran V.G. (2020). Unraveling Hematopoiesis through the Lens of Genomics. Cell.

[3] Pennington K.N., Taylor J.A., Bren G.D., Paya C.V. (2001). IκB Kinase-Dependent Chronic Activation of NF-κB Is Necessary for p21 WAF1/Cip1 Inhibition of Differentiation-Induced Apoptosis of Monocytes. Mol. Cell. Biol..

[4] Pagliara P., Lanubile R., Dwikat M., Abbro L., Dini L. (2005). Differentiation of monocytic U937 cells under static magnetic field exposure. Eur. J. Histochem..

[5] Chanput W., Mes J.J., Wichers H.J. (2014). THP-1 cell line: An in vitro cell model for immune modulation approach. Int. Immunopharmacol..

[6] Prasad A., Sedlářová M., Balukova A., Ovsii A., Rác M., Křupka M., Kasai S., Pospíšil P. (2020). Reactive Oxygen Species Imaging in U937 Cells. Front. Physiol..

[7] Prasad A., Kikuchi H., Inoue K.Y., Suzuki M., Sugiura Y., Sugai T., Tomonori A., Tada M., Kobayashi M., Matsue T. (2016). Simultaneous Real-Time Monitoring of Oxygen Consumption and Hydrogen Peroxide Production in Cells Using Our Newly Developed Chip-Type Biosensor Device. Front. Physiol..

[8] Chun E.M., Park Y.J., Kang H.S., Cho H.M., Jun D.Y., Kim Y.H. (2001). Expression of the apolipoprotein C-II gene during myelomonocytic differentiation of human leukemic cells. J. Leukoc. Biol..

[9] Yamamoto T., Sakaguchi N., Hachiya M., Nakayama F., Yamakawa M., Akashi M. (2008). Role of catalase in monocytic differentiation of U937 cells by TPA: Hydrogen peroxide as a second messenger. Leukemia.

[10] Zamani F., Shahneh F.Z., Aghebati-Maleki L., Baradaran B. (2013). Induction of CD14 Expression and Differentiation to Monocytes or Mature Macrophages in Promyelocytic Cell Lines: New Approach. Adv. Pharm. Bull..

[11] Mendoza-Coronel E., Castañón-Arreola M. (2016). Comparative evaluation ofin vitrohuman macrophage models for mycobacterial infection study. Pathog. Dis..

[12] Lund M.E., To J., O’Brien B.A., Donnelly S. (2016). The choice of phorbol 12-myristate 13-acetate differentiation protocol influences the response of THP-1 macrophages to a pro-inflammatory stimulus. J. Immunol. Methods.

[13] Kikuchi H., Prasad A., Matsuoka R., Aoyagi S., Matsue T., Kasai S. (2016). Scanning Electrochemical Microscopy Imaging during Respiratory Burst in Human Cell. Front. Physiol..

[14] Robinson J.M. (2008). Reactive oxygen species in phagocytic leukocytes. Histochem. Cell Biol..

[15] Pospíšil P., Prasad A., Rác M. (2014). Role of reactive oxygen species in ultra-weak photon emission in biological systems. J. Photochem. Photobiol. B Biol..

[16] Pospíšil P., Prasad A., Rác M. (2019). Mechanism of the Formation of Electronically Excited Species by Oxidative Metabolic Processes: Role of Reactive Oxygen Species. Biomolecules.

[17] Gutteridge J.M.C., Halliwell B. (2006). Free Radicals and Antioxidants in the Year 2000: A Historical Look to the Future. Ann. N. Y. Acad. Sci..

[18] Halliwell B., Gutteridge J. (2007). Free Radicals in Biology and Medicine.

[19] Radi R. (2018). Oxygen radicals, nitric oxide, and peroxynitrite: Redox pathways in molecular medicine. Proc. Natl. Acad. Sci. USA.

[20] Panieri E., Santoro M.M. (2016). ROS homeostasis and metabolism: A dangerous liason in cancer cells. Cell Death Dis..

[21] Schieber M., Chandel N.S. (2014). ROS Function in Redox Signaling and Oxidative Stress. Curr. Biol..

[22] Hawkins C.L., Davies M.J. (2001). Generation and propagation of radical reactions on proteins. Biochim. Biophys. Acta.

[23] Hawkins C.L., Davies M.J. (2019). Detection, identification, and quantification of oxidative protein modifications. J. Biol. Chem..

[24] Dean R.T., Fu S., Stocker R., Davies M. (1997). Biochemistry and pathology of radical-mediated protein oxidation. Biochem. J..

[25] Berlett B.S., Stadtman E.R. (1997). Protein oxidation in aging, disease, and oxidative stress. J. Biol. Chem..

[26] Davies M.J. (2003). Singlet oxygen-mediated damage to proteins and its consequences. Biochem. Biophys. Res. Commun..

[27] Di Mascio P., Martinez G.R., Miyamoto S., Ronsein G.E., Medeiros M.H.G., Cadet J. (2019). Singlet Molecular Oxygen Reactions with Nucleic Acids, Lipids, and Proteins. Chem. Rev..

[28] Kumar A., Prasad A., Sedlářová M., Pospíšil P. (2019). Organic radical imaging in plants: Focus on protein radicals. Free Radic. Biol. Med..

[29] Kumar A., Prasad A., Sedlářová M., Pospíšil P. (2019). Characterization of Protein Radicals in Arabidopsis. Front. Physiol..

[30] Mason R.P. (2004). Using anti-5,5-dimethyl-1-pyrroline N-oxide (anti-DMPO) to detect protein radicals in time and space with immuno-spin trapping. Free Radic. Biol. Med..

[31] Ramirez D.C., Mason R.P. (2005). Immuno-Spin Trapping: Detection of Protein-Centered Radicals. Curr. Protoc. Toxicol..

[32] Muñoz M.D., Gutierrez L.J., Delignat S., Russick J., Mejiba S.E.G., Lacroix-Desmazes S., Enriz R.D., Ramirez D.C., Gomez S.E., Enriz D.R. (2019). The nitrone spin trap 5,5-dimethyl-1-pyrroline N-oxide binds to toll-like receptor-2-TIR-BB-loop domain and dampens downstream inflammatory signaling. Biochim. Biophys. Acta (BBA)—Mol. Basis Dis..

[33] Augustyniak E., Adam A., Wojdyla K., Rogowska-Wrzesinska A., Willetts R., Korkmaz A., Atalay M., Weber D., Grune T., Borsa C. (2015). Validation of protein carbonyl measurement: A multi-centre study. Redox Biol..

[34] Verhoeckx K.C.M., Bijlsma S., de Groene E.M., Witkamp R.F., van der Greef J., Rodenburg R.J.T. (2004). A combination of proteomics, principal component analysis and transcriptomics is a powerful tool for the identification of biomarkers for macrophage maturation in the U937 cell line. Proteomics.

[35] Traore K., Sharma R., Thimmulappa R.K., Watson W.H., Biswal S., Trush M.A. (2008). Redox-regulation of Erk1/2-directed phosphatase by reactive oxygen species: Role in signaling TPA-induced growth arrest in ML-1 cells. J. Cell. Physiol..

[36] Stefanska J., Pawliczak R. (2008). Apocynin: Molecular Aptitudes. Mediat. Inflamm..

[37] Xie J., Hong E., Ding B., Jiang W., Zheng S., Xie Z., Tian D., Chen Y. (2020). Inhibition of NOX4/ROS Suppresses Neuronal and Blood-Brain Barrier Injury by Attenuating Oxidative Stress After Intracerebral Hemorrhage. Front. Cell. Neurosci..

[38] Barbieri S.S., Eligini S., Brambilla M., Tremoli E., Colli S. (2003). Reactive oxygen species mediate cyclooxygenase-2 induction during monocyte to macrophage differentiation: Critical role of NADPH oxidase. Cardiovasc. Res..

[39] Wardman P. (2007). Fluorescent and luminescent probes for measurement of oxidative and nitrosative species in cells and tissues: Progress, pitfalls, and prospects. Free Radic. Biol. Med..

[40] Gomez-Mejiba S.E., Zhai Z., Akram H., Deterding L.J., Hensley K., Smith N., Towner R.A., Tomer K.B., Mason R.P., Ramirez D.C. (2009). Immuno-spin trapping of protein and DNA radicals: “Tagging” free radicals to locate and understand the redox process. Free Radic. Biol. Med..

[41] Mason R.P. (2016). Imaging free radicals in organelles, cells, tissue, and in vivo with immuno-spin trapping. Redox Biol..

[42] Kiningham K.K., Cardozo Z.-A., Cook C., Cole M.P., Stewart J.C., Tassone M., Coleman M.C., Spitz D.R. (2008). All-trans-retinoic acid induces manganese superoxide dismutase in human neuroblastoma through NF-κB. Free Radic. Biol. Med..

[43] Kamiya T., Makino J., Hara H., Inagaki N., Adachi T. (2011). Extracellular-superoxide dismutase expression during monocytic differentiation of U937 cells. J. Cell. Biochem..

[44] Ximenes V.F., Kanegae M.P., Rissato S.R., Galhiane M.S. (2007). The oxidation of apocynin catalyzed by myeloperoxidase: Proposal for NADPH oxidase inhibition. Arch. Biochem. Biophys..

[45] Rőszer T. (2015). Understanding the Mysterious M2 Macrophage through Activation Markers and Effector Mechanisms. Mediat. Inflamm..

[46] Starr T., Bauler T., Malik-Kale P., Steele-Mortimer O. (2018). The phorbol 12-myristate-13-acetate differentiation protocol is critical to the interaction of THP-1 macrophages with Salmonella Typhimurium. PLoS ONE.

[47] Winterbourn C.C. (2008). Reconciling the chemistry and biology of reactive oxygen species. Nat. Chem. Biol..

[48] Ahmad A., Nawaz M.I., Siddiquei M.M., Abu El-Asrar A.M. (2021). Apocynin ameliorates NADPH oxidase 4 (NOX4) induced oxidative damage in the hypoxic human retinal Müller cells and diabetic rat retina. Mol. Cell. Biochem..

[49] Castor L.R.G., Locatelli K.A., Ximenes V.F. (2010). Pro-oxidant activity of apocynin radical. Free Radic. Biol. Med..

