# Supplementary material for: Free Radical-Mediated Protein Radical Formation in Differentiating Monocytes
Source: Int J Mol Sci. 2021 Sep 15;22(18):9963. doi: 10.3390/ijms22189963 (PMC8468151; doi:10.3390/ijms22189963)
Supplement: Supplementary file 1 [file ijms-22-09963-s001.zip › ijms-1356872-supplementary.pdf]

Supplementary Figure S1

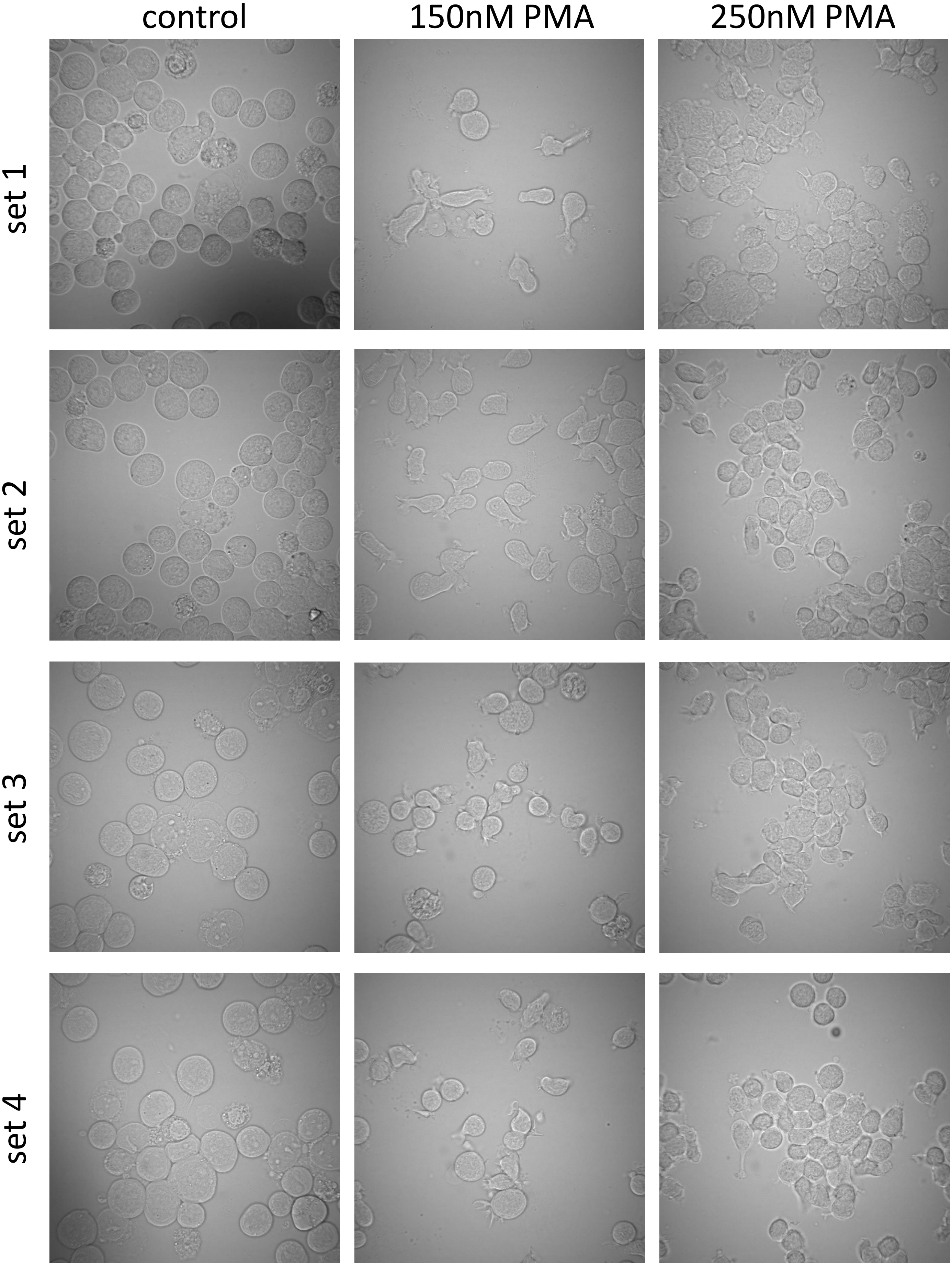

Supplementary Figure S1: Morphology of U937 cells in the absence (a) and presence of 150 nM PMA (b) and 250 nM PMA (c) for 72 h at 37° C.

Supplementary Figure S2

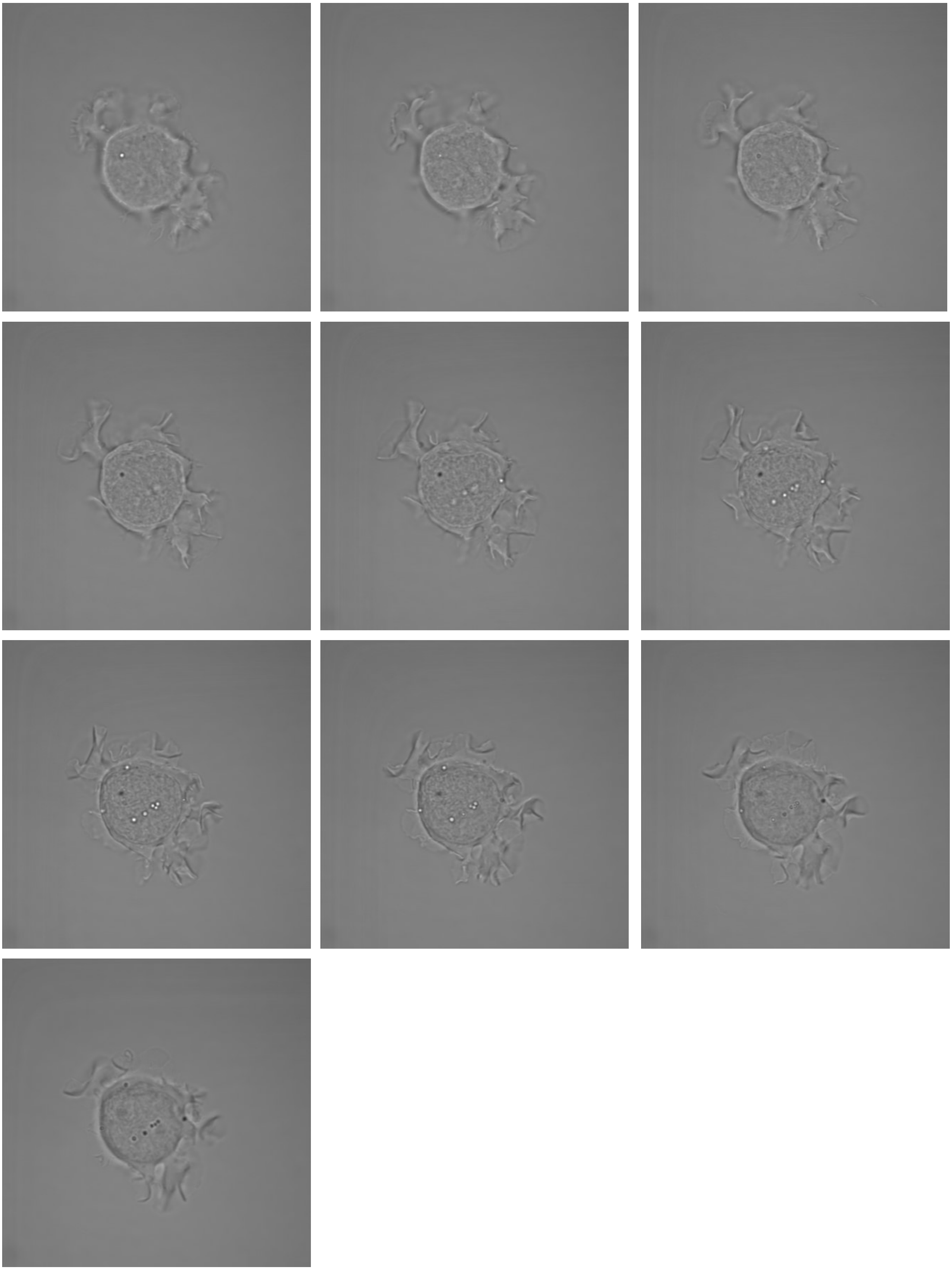

Supplementary Figure S2: Z-stack series of 10 optical sections (distance 0.48µm) through a differentiated U-937 cell treated with 150nM PMA for 48h.

Supplementary Figure S3

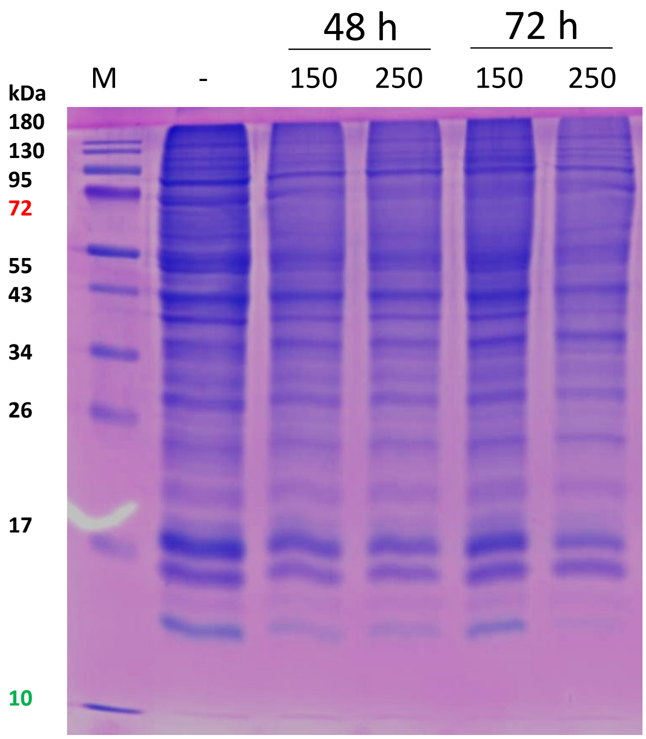

Supplementary Figure S3: Coomassie blue staining of cell homogenate treated with 150nM and 250nM PMA for 48 h and 72 h

Supplementary Figure S4

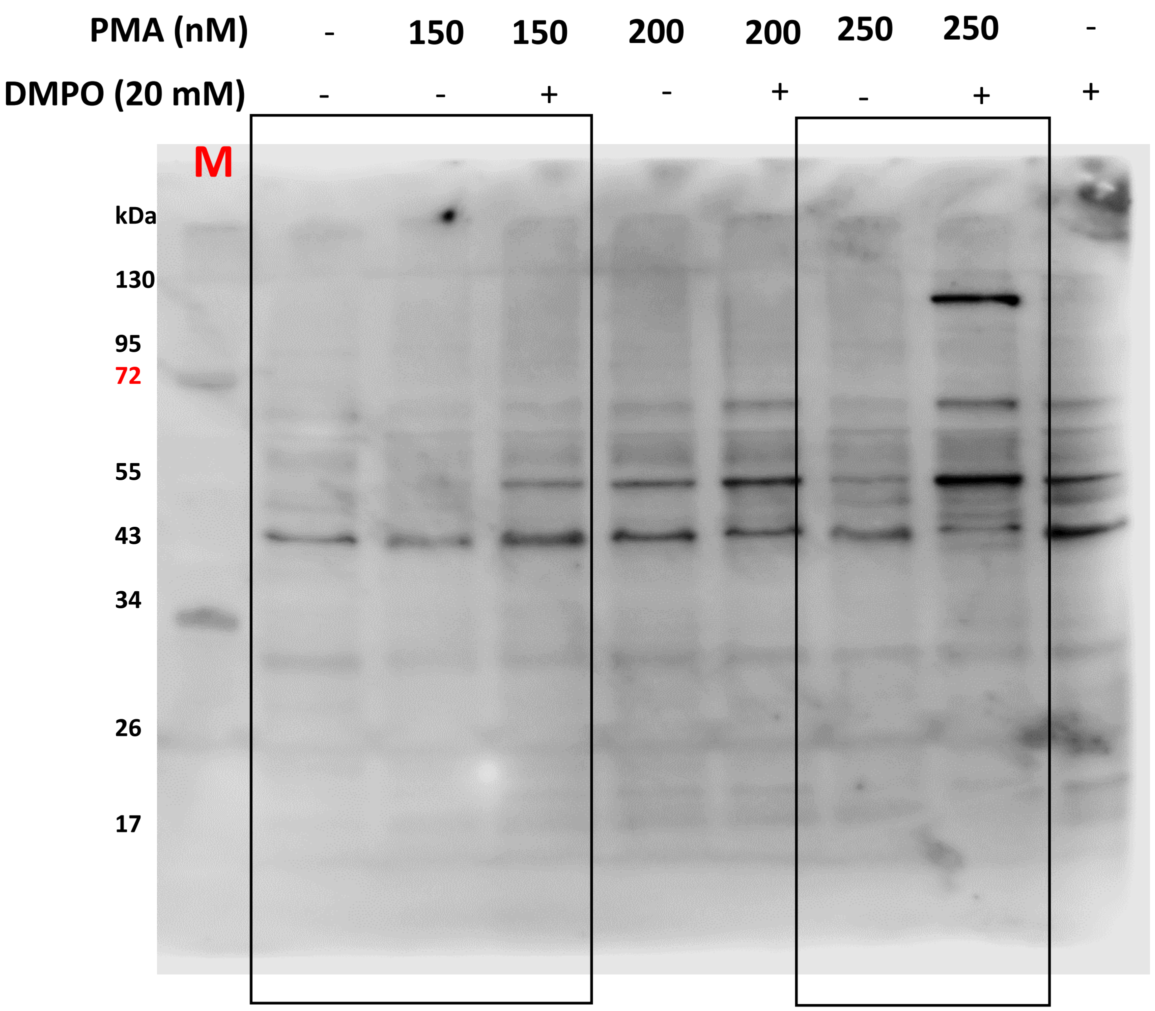

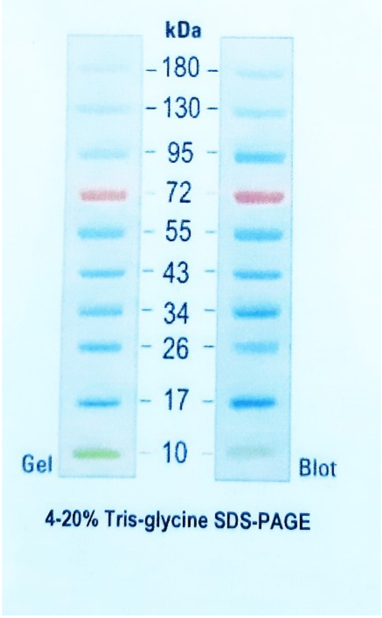

Prestained molecular weight marker

Supplementary Figure S4: Western blot analysis of Protein-DMPO nitrone adducts in whole cell homogenates treated with 150 nM, 200 nM and 250 nM of PMA for 72h. Lanes marked within the box are parts presented in the article. Lower panel shows the pre-stained molecular marker used.

Supplementary Figure S5

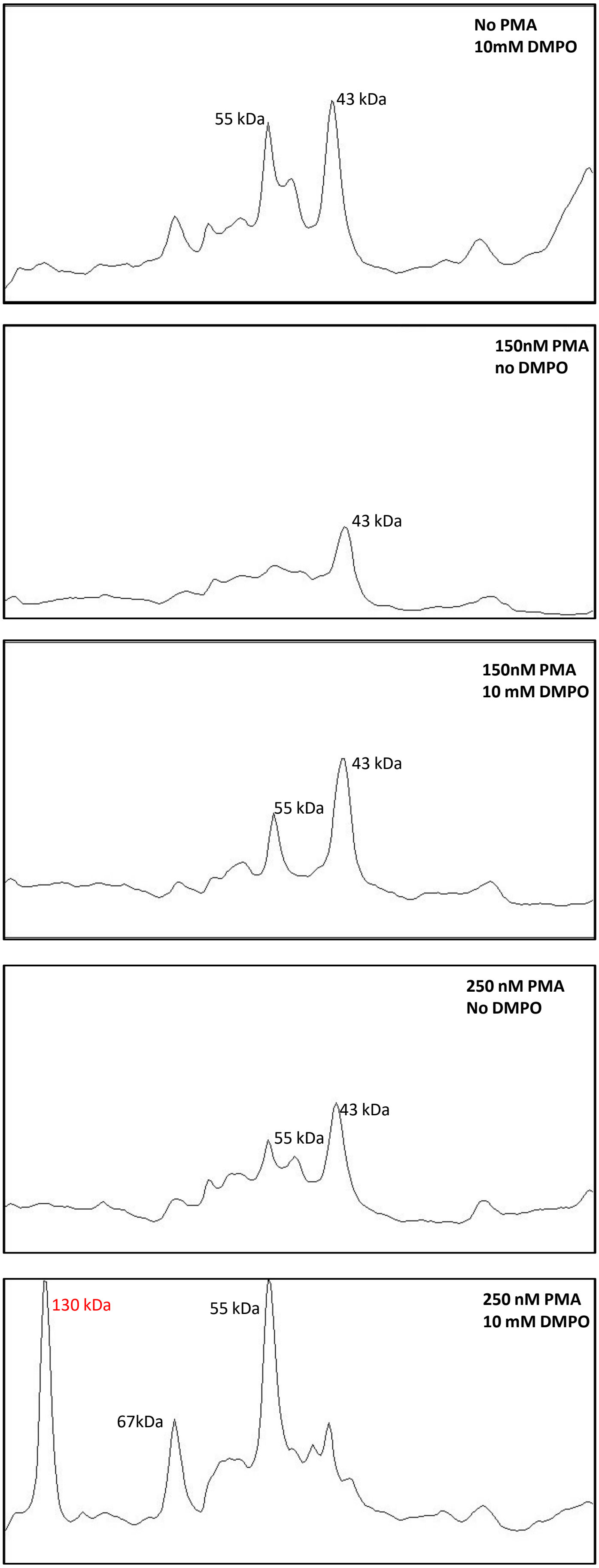

Supplementary Figure S5: Densitogram of protein expression pattern in PMA differentiated U-937 cells.

Supplementary Figure S6

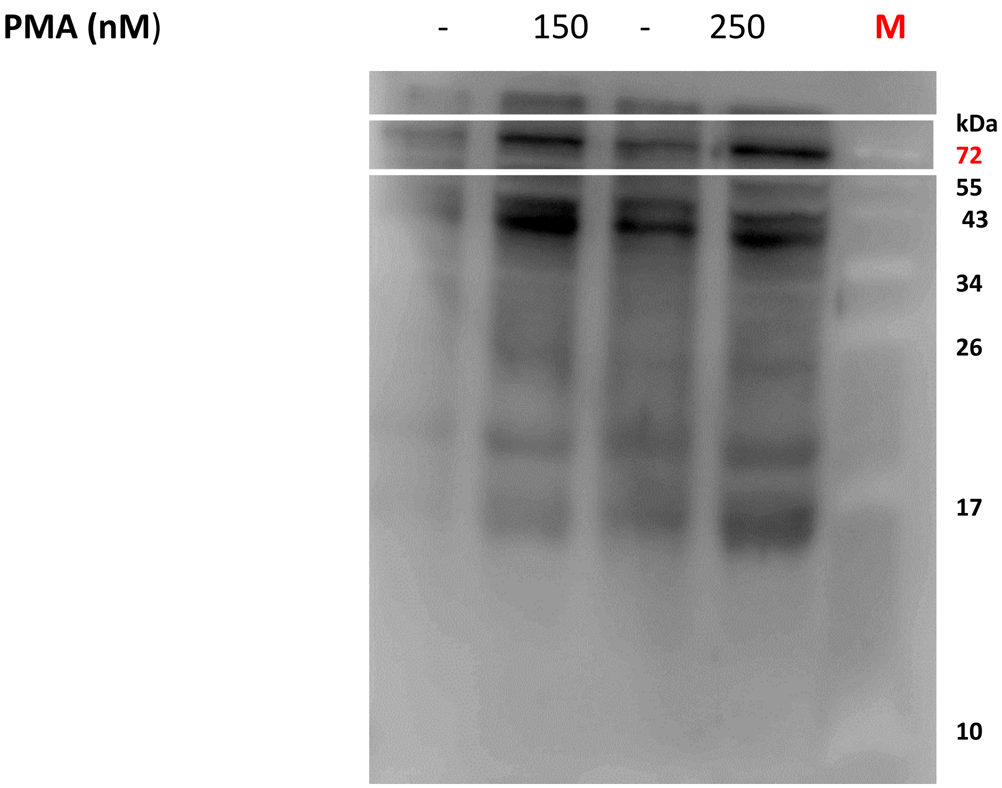

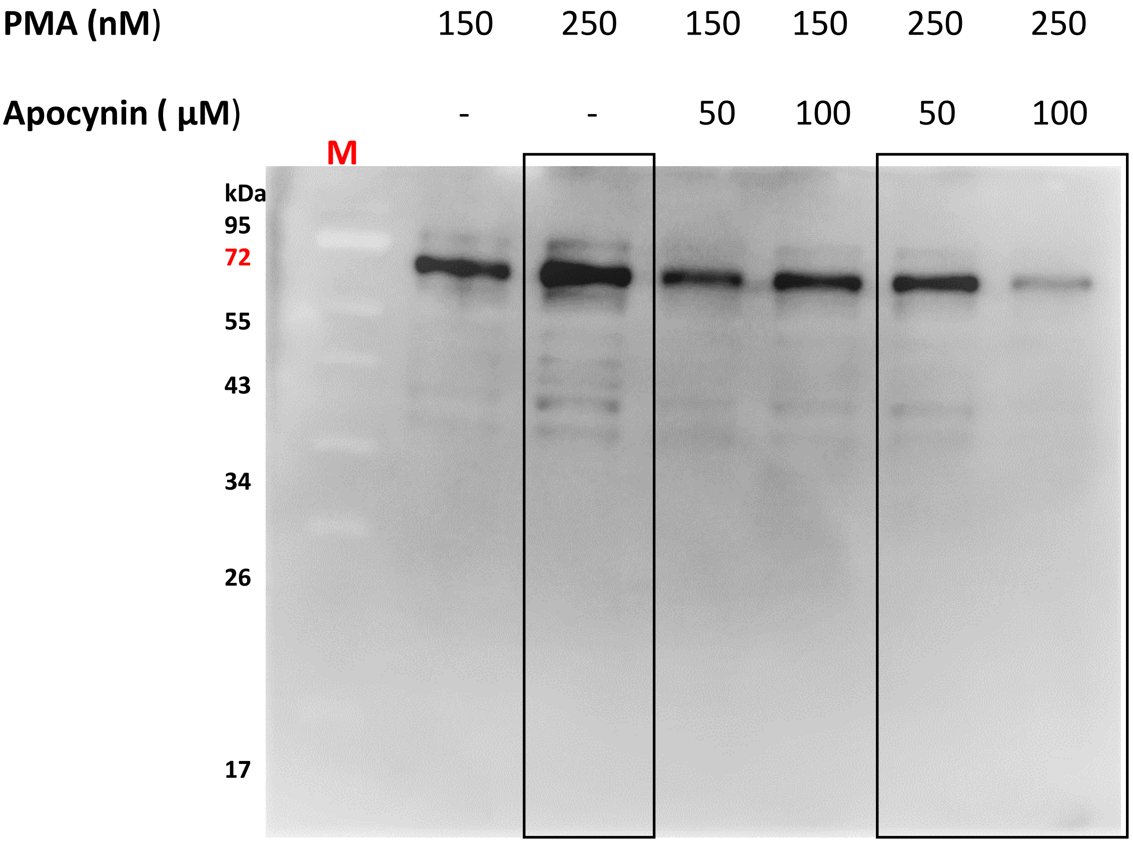

Supplementary Figure S6: NOX4 expression analysis in PMA (150nM and 250nM) activated macrophages: western blot analysis of NOX4 expression in whole-cell homogenates pre-treated with 50 µM and 100 µM Apocynin. Lanes marked within the box are parts presented in the article.
